# Editorial: Opioid-induced respiratory depression: neural circuits and cellular pathways

**DOI:** 10.3389/fphys.2023.1348910

**Published:** 2023-12-20

**Authors:** Astrid G. Stucke, Erica S. Levitt, Gaspard Montandon

**Affiliations:** ^1^ Department of Anesthesiology, Medical College of Wisconsin, Milwaukee, WI, United States; ^2^ Children’s Wisconsin, Milwaukee, WI, United States; ^3^ Department of Pharmacology, University of Michigan Medical School, Ann Arbor, MI, United States; ^4^ Department of Medicine, University of Toronto, Toronto, ON, Canada; ^5^ Department of Pharmacology and Toxicology, University of Toronto, Toronto, ON, Canada; ^6^ Keenan Research Centre for Biomedical Science, St. Michael’s Hospital, Toronto, ON, Canada

**Keywords:** opioid-induced respiratory depression, respiratory rate control, wooden chest syndrome (WCS), G protein regulation, prenatal opioid exposure

The analgesic and euphoric properties of the opium poppy were discovered thousands of years ago, and opiates and their derivatives have been widely used for pain management ever since. While opiates have beneficial properties such as analgesia, they also present side effects such as tolerance, addiction, sedation, and respiratory depression, which can be lethal in overdose. This latter side effect is due to the action of opioid drugs on their cognate opioid receptors, which are expressed in key respiratory regions of the brainstem. Opioids were first used to dissect the mechanisms controlling the timing of the respiratory phase (Palkovic et al., 2020; Ramirez et al., 2021). Opioid receptors have been found in multiple respiratory-related areas of the brainstem (Ding et al., 1996). Opioids activate postsynaptic receptors to hyperpolarize neurons in the Kölliker-Fuse nucleus (Levitt et al., 2015), the preBötzinger complex (Montandon et al., 2011), and premotor neurons (Bateman and Levitt, 2023). Opioids also decrease glutamate release presynaptic to respiratory neurons in the preBötzinger complex (Wei and Ramirez, 2019; Bateman and Levitt, 2023) and to premotor neurons (Bateman and Levitt, 2023). Other studies have used localized opioid receptor knock-out preparations (Bachmutsky et al., 2020; Varga et al., 2020) or localized injections of the opioid antagonist naloxone (Palkovic et al., 2021; Palkovic et al., 2022) to determine which brainstem areas are affected by clinically relevant opioid concentrations. Researchers have found that global opioid-induced respiratory depression (OIRD) resulted from a combined effect on the parabrachial nucleus/Kölliker-Fuse complex, the preBötzinger complex, the caudal medullary raphe, and possibly other areas supplying respiratory drive, and that the contribution of the individual areas depended on the opioid concentration.

More recently, the increasing number of fatal overdoses in patients with substance use disorders has spurred pragmatic clinical research to identify solutions to reverse and prevent OIRD. The animal studies included in this Research Topic focus on mechanisms of respiratory failure in different model organisms, such as rodents, goats, and dogs, and at different developmental stages, including prenatal, juvenile, and adult, bridging the gap between basic science and clinical research areas. These studies challenge the standard view that opioids uniformly reduce minute ventilation through a decrease in respiratory rate and that the effect size depends solely on the dose and kinetics of the respective agent. In this Research Topic, Neumueller et al. and Palkovic et al. highlight that opioids affect respiration with more inter-individual variability than previously described, i.e., intravenous opioids could also *increase* respiratory rate. This has been demonstrated in awake, freely behaving goats (Neumueller et al.) and in decerebrate dogs (Palkovic et al.), suggesting that the effect does not depend on behavioral state or opioid-induced itching. In decerebrate dogs, changes in phase timing and (fictive) tidal volume were associated with changes in the discharge frequency of inspiratory neurons in the pontine respiratory group and the preBötzinger complex, again highlighting the involvement of at least two areas of the central respiratory rhythm generator in OIRD.

Another phenomenon that has long been reported by clinicians but whose contribution to opioid-induced respiratory failure has not been systematically evaluated is the “Wooden chest syndrome” (Fahnenstich et al., 2000). In clinical practice, it is difficult to distinguish whether the observed inability to inflate the lungs after intravenous fentanyl administration is due to tonic activity of the intercostal and abdominal muscles or to glottic closure, i.e., laryngospasm. Three studies in this Research Topic provide new information on the muscles involved and the potential underlying neuronal mechanisms. Neuronal recordings in decerebrate dogs demonstrated that most inspiratory neuron types in the preBötzinger complex and parabrachial nucleus were depressed by intravenous remifentanil (Palkovic et al.), while many expiratory neuron types were not, possibly indicating a shift in balance toward expiratory muscle activation. In awake goats, intravenous fentanyl changed intermittent expiratory activity of the abdominal and intercostal muscles to strong tonic activity, while diaphragmatic activity remained phasic (Neumueller et al.). In addition, fentanyl evoked tonic genioglossus activity and phasic expiratory activity in addition to the baseline tonic discharge of the thyropharyngeus muscle. This increase in muscle tone would have resulted in airway narrowing and decreased airway and thoracic compliance. A similar observation was made in the more reduced *in situ* rat working heart-brainstem preparation (Cavallo et al.), where systemic opioids increased the tonicity of intercostal muscle activity, indicative of decreased thoracic compliance. In this study, opioids also caused tonic diaphragm activity, which has not been described in goats and would have further reduced ventilatory compliance. In addition, opioid effects on the upper airways were more aligned to relaxation of laryngeal muscles, similar to that observed with neuromuscular paralysis, rather than constriction. This would still result in a loss of upper airway function regulating airflow. Cavallo et al. also showed that effects on diaphragm and intercostal muscle activity correlated with the intrinsic efficacy of the agonist.

There are many potential second messenger pathways downstream of mu-opioid receptors that could mediate the respiratory effects of opioids, including in particular G proteins and arrestins. Heterotrimeric G proteins consisting of α and βγ subunits are the most proximal to the mu-opioid receptor, and the activity of G proteins is negatively regulated by Regulators of G protein Signaling (RGS) proteins. Danaf et al. showed that the respiratory rate depression resulting from dialysis of the mu-opioid receptor agonist DAMGO into the preBötzinger complex was mediated by βγ G protein subunits. Blockade of these βγ subunits reversed DAMGO-induced respiratory rate depression but had no effect alone. In contrast, inhibition of RGS4 alone depressed breathing rate, suggesting that enhancing G protein signaling in the preBötzinger Complex could depress breathing, but RGS4 did not mediate OIRD.

Infants born to mothers who use opioids may experience neonatal abstinence syndrome. To explore the impact of prenatal opioid exposure on the development central respiratory control networks in neonates, Beyeler et al. treated pregnant mouse dams with methadone daily from embryonic day 17 to postnatal day 5. Respiratory motor output in brainstem-spinal cord preparations was impaired immediately after birth in pups with prenatal opioid exposure, with evidence of lingering methadone effects and the development of short-lived tolerance. Prenatal methadone had a more lasting effect on the respiratory pattern, with reduced breath-to-breath variability observed until postnatal day 5. These effects of opioids on the developing respiratory network likely occur outside the preBötzinger complex since the fictive respiratory motor output from isolated rhythmic slices was unchanged.

A better understanding of OIRD will ultimately improve patient care. The articles in this Research Topic highlight how this research benefits from the use of multiple animal species and a range of experimental paradigms. Continued research is necessary to integrate the knowledge from receptor signaling to respiratory muscle output, including in subjects of different ages, different behavioral states, and after chronic opioid use, to gain a more complete understanding of the spectrum of opioid effects on breathing.
